# Automated Detection and Classification of Desmoplastic Reaction at the Colorectal Tumour Front Using Deep Learning

**DOI:** 10.3390/cancers13071615

**Published:** 2021-03-31

**Authors:** Ines P. Nearchou, Hideki Ueno, Yoshiki Kajiwara, Kate Lillard, Satsuki Mochizuki, Kengo Takeuchi, David J. Harrison, Peter D. Caie

**Affiliations:** 1 Quantitative and Digital Pathology, School of Medicine, University of St Andrews, St Andrews KY16 9TF, UK; david.harrison@st-andrews.ac.uk (D.J.H.); pdc5@st-andrews.ac.uk (P.D.C.); 2 Department of Surgery, National Defense Medical College, 3-2 Namiki, Tokorozawa, Saitama 359-8513, Japan; ueno_surg1@ndmc.ac.jp (H.U.); ykaji@ndmc.ac.jp (Y.K.); s-mochi@ndmc.ac.jp (S.M.); 3 Indica Labs, Inc., 2469 Corrales Rd Bldg A-3, Corrales, NM 87048, USA; kate@indicalab.com; 4 Division of Pathology, Cancer Institute, Japanese Foundation for Cancer Research, 3-8-31 Ariake, Koto, Tokyo 135-8550, Japan; kentakeuchi-tky@umin.net; 5 Department of Pathology, Cancer Institute Hospital, Japanese Foundation for Cancer Research, 3-8-31 Ariake, Koto, Tokyo 135-8550, Japan; 6 Pathology Project for Molecular Targets, Cancer Institute, Japanese Foundation for Cancer Research, 3-8-31 Ariake, Koto, Tokyo 135-8550, Japan

**Keywords:** deep learning, image analysis, desmoplastic reaction, colorectal cancer, digital pathology

## Abstract

**Simple Summary:**

Desmoplastic reaction (DR) has previously been shown to be a promising prognostic factor in colorectal cancer (CRC). However, its manual reporting can be subjective and consequently consistency of reporting might be affected. The aim of our study was to develop a deep learning algorithm that would facilitate the objective and standardised DR assessment. By applying this algorithm on a CRC cohort of 528 patients, we demonstrate how deep learning methodologies can be used for the accurate and reproducible reporting of DR. Furthermore, this study showed that the prognostic significance of DR was superior when assessed through the use of the deep learning classifier than when assessed manually. In this study, we demonstrate how the application of machine learning approaches can help by not only identifying complex patterns present within histopathological images in a standardised and reproducible manner, but also report a more accurate patient stratification.

**Abstract:**

The categorisation of desmoplastic reaction (DR) present at the colorectal cancer (CRC) invasive front into mature, intermediate or immature type has been previously shown to have high prognostic significance. However, the lack of an objective and reproducible assessment methodology for the assessment of DR has been a major hurdle to its clinical translation. In this study, a deep learning algorithm was trained to automatically classify immature DR on haematoxylin and eosin digitised slides of stage II and III CRC cases (*n* = 41). When assessing the classifier’s performance on a test set of patient samples (*n* = 40), a Dice score of 0.87 for the segmentation of myxoid stroma was reported. The classifier was then applied to the full cohort of 528 stage II and III CRC cases, which was then divided into a training (*n* = 396) and a test set (*n* = 132). Automatically classed DR was shown to have superior prognostic significance over the manually classed DR in both the training and test cohorts. The findings demonstrated that deep learning algorithms could be applied to assist pathologists in the detection and classification of DR in CRC in an objective, standardised and reproducible manner.

## 1. Introduction

Colorectal cancer (CRC) is one of the most common cancers worldwide [1] and is currently staged according to the tumour, node, metastasis (TNM) staging system [2]. It is now well established that the tumour microenvironment (TME) contributes to tumour aggressiveness and patient survival [3,4]. Desmoplastic reaction (DR) or desmoplasia, refers to the presence of excessive extracellular matrix at the invasive tumour front [5]. Previous studies have shown that DR is associated with adverse clinicopathological findings in CRC such as advanced T stage, lymphatic or venous invasion and poor clinical outcome [5,6,7]. 

A 3-tier classification system for the categorisation of DR has previously been shown to be promising in the stratification of CRC patients into high-, mid- and low-risk of disease-specific death [5,6,7]. This system requires the identification and assessment of all haematoxylin and eosin (H&E) slides, generated per patient, and which contain any invasive tumour front beyond the muscular layer. DR is then categorised into immature, intermediate or mature based on the presence of myxoid stroma or keloid-like collagens. Patients with immature DR were shown to have the worst disease-specific survival rate followed by patients with intermediate DR, whereas patients with mature DR were shown to confer the best disease-specific survival rate [6,7].

Although previous studies demonstrated that this 3-tier classification system can be promising in the stratification of CRC patients into prognostic subgroups, this method requires the assessment of multiple slides. Previously, we developed a more efficient single-slide method where only the slide containing the deepest tumour invasion was examined for DR. The single-slide method results were shown to be highly correlated with the results of the currently used multi-slide method. Moreover, the prognostic significance of DR when assessed on a single slide was almost identical to the results from the multi-slide method [7]. Therefore, assessment of the slide containing the deepest portion of the invasive front was shown to be sufficient for the stratification of CRC patients according to DR.

Even though assessment of DR on a single slide might reduce the time and effort required by the pathologist, the interobserver agreement is suboptimal. Several factors can influence the degree of interobserver variability such as the stain colour variation. Although the interobserver agreement reported in previous studies was within acceptable ranges [7,8,9], concerns regarding its reproducible reporting is one of the main barriers to its clinical translation. Previously, through the use of machine learning and automated image analysis, we have successfully automatically quantified other features present at the CRC invasive front, such as tumour budding [10], lymphocytic and macrophage infiltration [4] and poorly differentiated clusters [11]. However, in those studies, multiplex immunofluorescence was used to label these specific features of interest and hence threshold-based algorithms in combination with shallow machine learning approaches were used for their classification. DR, however, is not a specific feature but a pattern which needs to be recognised and categorised on H&E slides. Deep learning algorithms have previously been shown to be promising in identifying patterns on H&E whole-slide images [12,13,14,15]. In the present study, we aimed to develop a deep learning algorithm for the automatic DR classification on a single H&E slide containing the deepest tumour invasion of each patient. We further aimed to assess and compare the prognostic significance DR assessed through the deep learning algorithm and by eye.

## 2. Results

### 2.1. Patients’ Characteristics

This study included a training cohort of 396 patients, of which 159 were female and 237 were male. The test set included 132 patients of which 54 were female and 78 were male. Two hundred and six stage II and 190 stage III patients were included in the training cohort, whereas 74 stage II and 58 stage III patients were included in the test set. Ninety-four patients were found to have immature DR within the training cohort, whereas 31 patients with immature DR were included within the test set. The full clinicopathological characteristics of the training and test sets are shown in Table 1.

### 2.2. Classifier Accuracy Evaluation

**Figure 1:**
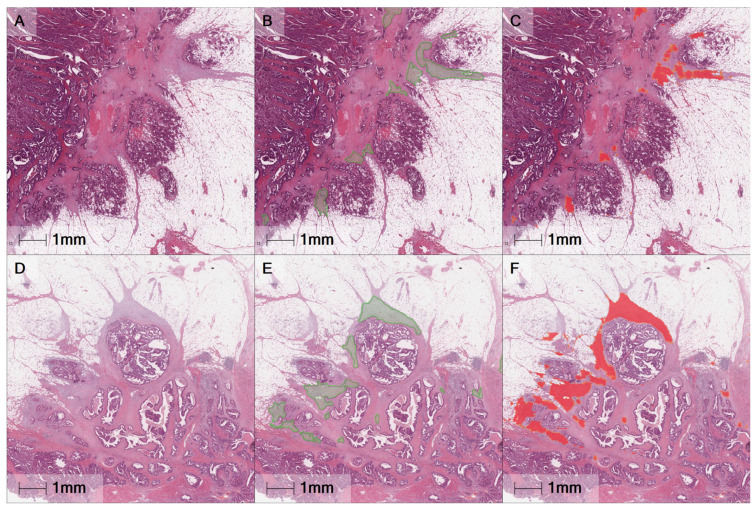
Examples of automatic desmoplastic reaction detection on annotated slides with myxoid stroma. (**A**,**D**) H&E-stained slides with myxoid stroma; (**B**,**E**) Manually annotated regions of myxoid stroma shown in green; and (**C**,**F**) Image analysis mask, automatically classified myxoid stroma regions shown in red.

A single digitised H&E slide, selected from all slides per case, containing the deepest portion of the invasive front, from all cases within the study, was manually selected and categorised into immature or other DR type based on the presence of myxoid stroma in the extramural tumour front. A DenseNet neural network, integrated within HALO^®^ AI was then trained to segment myxoid stroma from non-myxoid stroma areas using 41 cases. The classifier’s accuracy was assessed on 40 unseen cases, 20 immature DR cases (containing annotated myxoid stroma), and 20 other DR type cases. The Dice score was then used in order to assess the performance of the classifier and results showed that the classifier achieved a Dice score of 0.87 in segmenting myxoid stroma. Examples of images with manual annotations and automatically segmented myxoid stroma areas are shown in Figure 1.

### 2.3. Automated DR Classification

**Figure 2:**
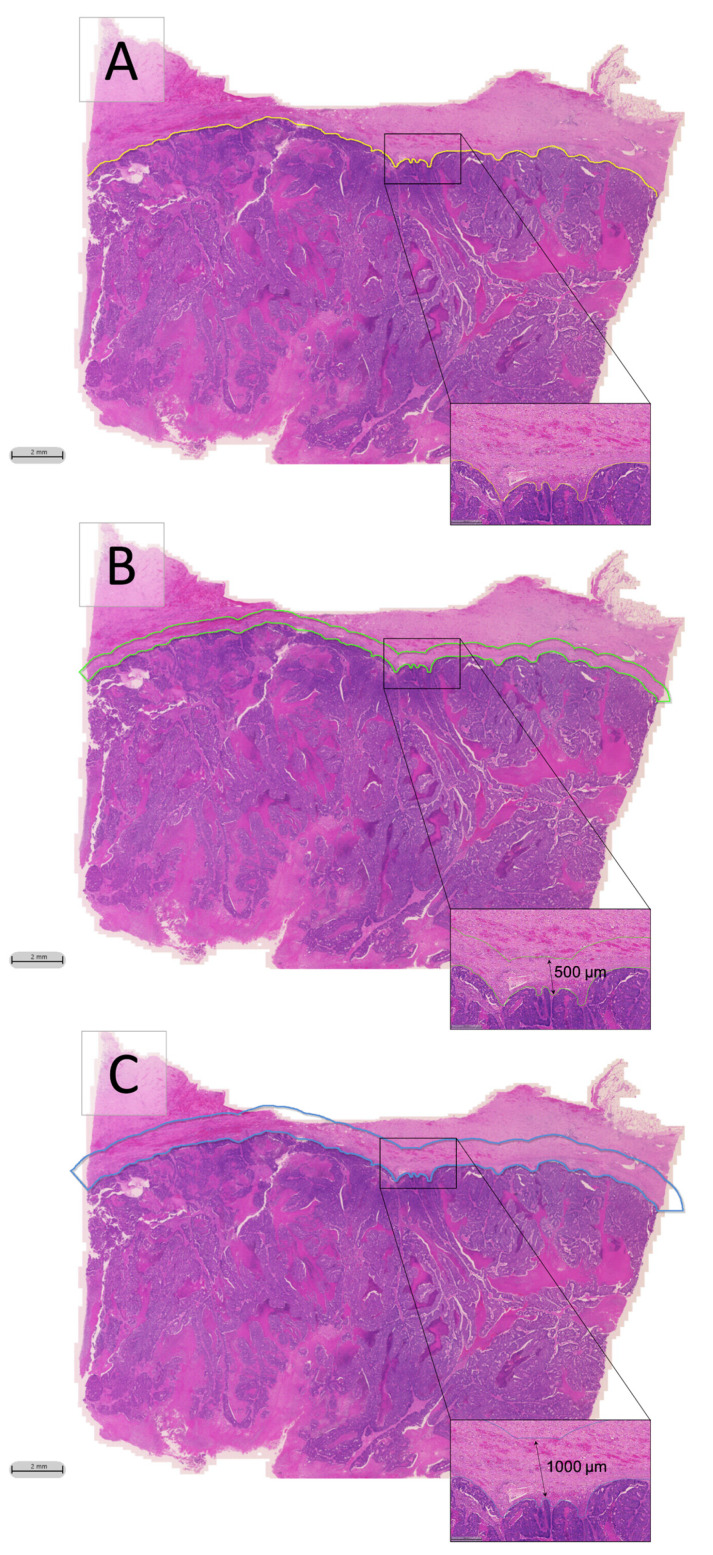
Desmoplastic reaction regions of interest. (**A**) Annotated tumour front, shown in yellow; (**B**) Desmoplastic reaction Margin 1 shown in green, being a 500 μm border from the invasive front; and (**C**) Desmoplastic reaction Margin 2 shown in blue, being a 1000 μm border from the invasive front.

The classifier was then applied to the remaining patient samples, so that all cases (*n* = 528) had been automatically analysed for the detection of myxoid stroma. Specifically, the classifier was run across 2 automatically created regions of interest, within a width span of either 500 or 1000 μm outwards from the manually delineated tumour’s invasive front (Margin 1 and Margin 2, respectively; Figure 2). The total myxoid stroma area, the average myxoid stroma area, and the largest single myxoid stroma area present within these regions were automatically detected. These features were categorised according to optimal cut-off points obtained through survival analysis of the training cohort data (*n* = 396). However, the largest single myxoid stroma area was also categorised according to the literature-dictated cut-off point [6,7,16] (0.196 mm^2^) for comparison. Since the largest single myxoid stroma area was categorised according to two cut-off points, the automatically derived value is referred to as the automatic cut-off point (ACP) whereas the one used when manually assessing for DR is referred to as the manual cut-off point (MCP). All features and their cut-off points are shown in Table 2. Cases with feature values greater than the cut-off points were regarded as having immature DR, whereas patients with feature values less than the cut-off points were regarded as having other DR type.

### 2.4. Survival Analysis

**Figure 3:**
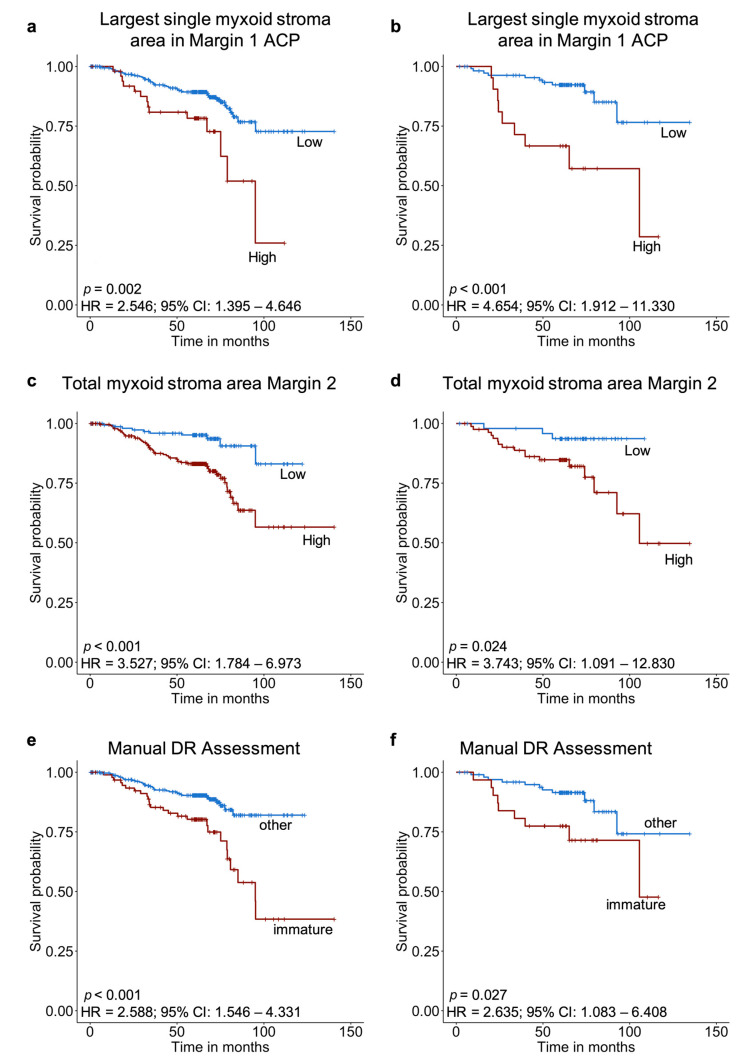
Kaplan–Meier survival analysis for the largest single myxoid stroma area in Margin 1 ACP, total myxoid stroma area Margin 2, and manual DR Assessment for the training and test sets. (**a**) Largest single myxoid stroma area in Margin 1 ACP for the training set, (**b**) largest single myxoid stroma area in Margin 1 ACP for the test set, (**c**) total myxoid stroma area Margin 2 for the training set, (**d**) total myxoid stroma area Margin 2 for the test set, (**e**) manual DR Assessment for the training set, and (**f**) manual DR Assessment for the test set. *p* values from KM analysis. HR and CI from univariate Cox regression analysis.

Univariate Cox regression was initially applied to the training set in order to assess the prognostic significance of T stage, N stage, differentiation, tumour type, automatically assessed DR features and manually assessed DR. Results showed that all of the features except differentiation were prognostically significant with hazard ratios varying between 1.792 and 3.527 (Table 3). Multivariate Cox regression with a forward stepwise method was then applied in order to compare the prognostic significance of the features and to identify the most prognostically significant factors. The total myxoid stroma area in Margin 2 was shown to be the strongest prognostic factor (HR = 3.527; 95% CI, 1.784–6.973; *p* < 0.001), followed by pN stage (HR = 1.490; 95% CI, 1.062–2.091; *p* = 0.021, Table 4). Manually assessed DR was not found to be significant when multivariate Cox regression was applied. KM survival analysis was also performed in order to identify the feature (from all DR-related features) which stratified the training cohort with the greatest accuracy. Results showed that the largest single myxoid stroma area in Margin 1 binarised using the ACP could stratify the training cohort with the greatest accuracy (Figure 3). Specifically, patients with a single myxoid stroma area larger than 1.04863 mm^2^ in Margin 1 were shown to confer significantly worse disease-specific survival (26.0% survival rate) than patients with a single myxoid stroma of a smaller area (72.7% survival rate, Figure 3a). KM survival analysis also showed that the total myxoid stroma area in Margin 2 previously selected by the multivariate Cox regression was significantly associated with disease-specific survival. Patients with a higher total myxoid stroma area than 0.31949 mm^2^ in Margin 2 were shown to confer significantly worse survival outcome (56.6% survival rate) than patients with lower total myxoid stroma area than this value (83.1% survival rate, Figure 3c). Finally, KM analysis showed that patients with immature DR conferred worse disease-specific survival (38.2% survival rate) than patients with other stroma types (82.0% survival rate, Figure 3e) when assessed using the manual classification method. 

The 2 most prognostically significant DR-related features identified from the training set (largest single myxoid stroma area in Margin 1 ACP and total myxoid stroma area in Margin 2) as well as the manually assessed DR, were assessed on the test set for survival analysis. The values of the cut-off points for the features derived from the training set were directly applied to the test set. Univariate Cox regression showed that all 3 features had high prognostic significance (largest single myxoid stroma area in Margin 1 ACP: HR = 4.654; 95% CI, 1.912–11.330; *p* < 0.001, total myxoid stroma area in Margin 2: HR = 3.743; 95% CI, 1.091–12.830; *p* = 0.036, and manually assessed DR: HR = 2.635; 95% CI, 1.083–6.408; *p* = 0.033; Table 5). KM analysis was further applied and results showed that all 3 features were significantly associated with disease-specific survival. However, the prognostic significance of the automatically detected DR was superior. Specifically, the use of the automatically calculated size cut-off for the largest single myxoid stroma area outperformed the manual one that used the area of the size of the microscopic field of a 40× objective lens. Furthermore, the largest single myxoid stroma area in Margin 1 binarised using the ACP achieved the most significant patient stratification. Patients with a single myxoid stroma area larger than 1.04863 mm^2^ in Margin 1 were shown to confer significantly worse disease-specific survival (28.6% survival rate) than patients with a single myxoid stroma of a smaller area (76.6% survival rate, Figure 3b). Similarly, patients with a higher total myxoid stroma area than 0.31949 mm^2^ in Margin 2 were shown to confer significantly worse survival outcome (49.7% survival rate) than patients with lower total myxoid stroma area than this value (93.7% survival rate, Figure 3d). Finally, patients with immature DR were shown to confer worse disease-specific survival (47.6% survival rate) than patients with other DR types (74.2% survival rate, Figure 3f) when assessed manually.

## 3. Discussion

A wealth of studies has previously aimed to understand the complex tumour–stroma interactions and their association with patient clinical outcome [17,18]. DR, referring to the excessive fibrous tissue formation surrounding the tumour, has been shown to be highly prognostic in CRC. Specifically, immature DR, categorised based on the presence of large areas of myxoid stroma at the extramural tumour front, has repeatedly been associated with adverse clinicopathological features and poor disease-specific and relapse-free survival [5,6,16]. In the methodology currently presented in the literature, DR categorisation is performed manually on all tumour slides containing any invasive front beyond the muscular layer [19]. However, we have previously shown that DR can be assessed on a single tissue slide containing the tumour deepest invasive front [7]. Although this approach is more time-efficient, it does not guarantee the reporting of standardised and reproducible results.

In this study, we have developed a deep learning-based methodology to automatically categorise DR present at the extramural tumour front on a single tissue section containing the deepest advancing edge of the tumour. The deep learning classifier was shown to have high accuracy in detecting myxoid stroma when assessed on unseen cases with a Dice score of 0.87. The DR categorisation using the results from the automated deep learning classifier was shown to have high prognostic significance both on a training and a test set of CRC patient samples. Furthermore, the automatic DR categorisation was found to be superior to the manual DR categorisation in patient stratification.

The accuracy of the algorithm to identify myxoid stroma was assessed on an unseen set of images and this evaluation translated into significant survival statistics when the algorithm was applied to identify myxoid stroma across the entire patient cohort. When comparing the prognostic significance of the automatically classed DR and the manual assessment of DR, the automatically classed DR was shown to have superior prognostic significance. This difference in the prognostic significance can be explained by the discrepancies present within the 2 methods. During its manual assessment, DR is categorised as immature if the myxoid stroma is larger than a microscopic field of a 40× objective lens. Specifically, the entire area of the microscopic field needs to be taken up by the myxoid stroma. Cases where myxoid stroma of a thin and elongated shape, which could be of an area greater than a microscopic field of a 40× objective lens, and yet does not take up the entire field of view, would not be classed as immature DR. Moreover, although this cut-off point can be easily applied across institutes due to use of a standard 40× objective microscope lens, interobserver agreement is suboptimal. The use of the deep learning classifier and image analysis allows for the standardised and reproducible DR assessment but also for the reporting of the exact size of every myxoid stroma area. This can, therefore, result in the quantitative DR reporting and in the identification of optimal cut-off points for the classification of the DR as immature. Furthermore, this classifier not only reports the size of the myxoid stroma but also the total myxoid stroma area present at the extramural tumour front. The manual reporting of the total myxoid stroma area present in each slide would be an extremely hard task to regularly perform as it is labour-intensive, time-consuming and subjective. In fact, to the best of our knowledge, there has not been any study assessing the prognostic significance of the total myxoid stroma area present at the extramural tumour front. This study is therefore the first to demonstrate that this feature had high prognostic significance in CRC when assessed both on a training and a test set. Finally, as previously mentioned, H&E staining variability can contribute to the sub-optimal inter-observer agreement when assessing for DR. Although stain variation is also a well-known issue in image analysis, several techniques have been proposed to overcome this. One such technique is colour augmentation, which simulates a variety of stain variations throughout the training of the classifier in order to produce stain-invariant classifiers [20]. Indeed, the deep learning algorithm used in this study included such a colour augmentation step, as previously applied by Liu et al. [14], which addresses the issue of colour variation and thus could be advantageous over manual DR assessment.

Previous studies have shown that the prognostic significance of DR outperformed that of other conventional prognostic factors used in international guidelines such as T stage [5,21]. This is of particular interest in stage II CRC, where about 20–30% of the patient population experiences disease recurrence or poor clinical outcome [22,23]. Currently, a randomised controlled study regarding the use of adjuvant chemotherapy in stage II CRC is being conducted in Japan. The target sample size of this study is 1680 patients and the patient recruitment criteria are based on 4 pathological factors which include DR categorisation [24]. Should the results of this study show that DR categorisation is highly significant in identifying patients who would benefit from adjuvant therapy, its clinical translation might be recommended. Modern pathology is moving toward a digital workflow [25] evident by the Food and Drug Administration (FDA) clearance of the first whole-slide imaging system for primary diagnostics in 2017 [26] and the initiation of proceedings to undergo full digitisation of the National Health Service (NHS) Greater Glasgow and Clyde in Scotland [27]. Given that standardisation and reproducibility are key in diagnostic tests as well as patient care [28], the clinical translation of classifiers, such as the deep learning system for the categorisation of DR proposed here, might prove to be very promising. The advantages of an automated system would negate any inter-observer variability and ensure the reliability and reproducibility of the results, but also could reduce the workload of the pathologists while providing the potential to report results quickly due to continuous analysis workflows. 

Deep learning algorithms have shown promise in patient diagnosis and prognosis across multiple cancers [12,13,14,15]. Most of these algorithms require the break-up of the tissue architecture into small patches in order to successfully train and analyse the sample by deep learning. The deep learning algorithm used in this study has the advantage of being able to be directly applied on whole-slide images instead of small image patches. This therefore negates the need of image reduction and patch extraction following the digital image acquisition. Moreover, machine learning algorithms involve a number of hyperparameters that can be tuned to achieve maximal classifier performance. Here, we aimed to use a widely applicable, ready-to-deploy algorithm, and therefore, the default values of hyperparameters that were specified within the commercially available DenseNet neural network from Indica Labs were used. Unlike other studies, here there was no need to generate a bespoke algorithm in, e.g., python or similar software. The results of this study show the promise of validating an “off the shelf” deep learning architecture. Further, it opens up this powerful research methodology to institutes that may not have access to data scientists and the expertise to generate the complicated programming, previously needed to apply this technology to the field of pathology.

Our work also has some limitations. First, the algorithm was trained on a limited set of whole-slide images. Second, the images used to train and validate the performance of the algorithm originated from a single centre. Although the performance of the algorithm was shown to be significant on the unseen cases, the inclusion of additional multi-institutional images, both for the training and the validation step of the algorithm, would increase the robustness of the classifier and aid in its clinical translation. Finally, the manual DR assessment involves the categorisation of DR into 3 types, those being the immature, intermediate and mature type. However, in this study, DR was only classed as immature or other DR type. This was due to the absence of any guidelines regarding the required size of the keloid-like collagens to be classed as intermediate DR. Furthermore, as the immature DR has been previously correlated to the worst survival, this was also seen as the most important feature to be able to classify. However, pending further clarification of the required keloid-like collagen size, an additional class could be added to the algorithm and hence this classifier might prove to be also promising in categorising the DR into 3 types instead of 2.

## 4. Materials and Methods

### 4.1. Patient Material

This study included an initial cohort of 280 stage II and 248 stage III CRC patients who had undergone surgical resection at the National Defense Medical College Hospital, Japan, over the years 2006–2011. Associated clinicopathological data such as age, gender and pT stage were taken from the original pathology report. Patient follow-up was up to 11.2 years. The initial cohort was split into a training cohort which included 75% of the patients (*n* = 396) and a test cohort which included 25% of the patients (*n* = 132). This was performed using stratified sampling based on both clinical outcome and DR category. Clinicopathological characteristics of these cohorts are summarised in Table 1. This study was approved by the Ethics Committee of the National Defense Medical College (approval ref: No. 2992). Further ethical clearance was not required as the acquired data were anonymised.

### 4.2. Manual Histologic Evaluation of Desmoplastic Reaction

A single H&E glass slide containing the deepest portion of the invasive front was manually selected from all diagnostic slides for each patient by I.P.N. and Y.K. This slide was then digitised using a Leica Aperio AT2 whole-slide scanner (Leica Biosystems, Vista, CA, USA) with a 40× objective. According to the 3-tier classification system previously proposed [6], the presence of myxoid stroma greater than a microscopic field of a 40× objective lens (0.196 mm^2^) beyond the muscularis propria is classed as immature DR. Those samples with a presence of keloid-like collagens and absence of myxoid stroma greater than 0.196 mm^2^ at the extramural tumour front are classed as intermediate DR. However, due to the absence of any guidelines regarding the size of keloid-like collagens required to be classed as intermediate DR, this category was abandoned in this study. Therefore, here, DR was categorised into “immature DR type” based on the presence of myxoid stroma greater than a microscopic field of a 40× objective lens (0.196 mm^2^) beyond the muscularis propria or “other DR type” based on the absence of size significant myxoid stroma at the extramural tumour front. 

### 4.3. Training the Deep Learning Classifier

Digitised whole-slide images of the slide containing the deepest portion of the invasive front were uploaded into HALO^®^ software (Indica Labs, Inc., Corrales, NM, USA) for the image analysis process. Forty-one cases were randomly selected for the training of the classifier. These included 37 immature DR type cases (stage II: *n* = 19 and stage III: *n* = 18) and 4 stage II cases that contained myxoid stroma but not of an area greater than a microscopic field of a 40× objective lens (thus they would be classified as “other DR type”). Myxoid stroma areas present on these 41 slides were jointly annotated by I.P.N. and H.U. within the HALO^®^ software. These were used to train a DenseNet neural network, integrated within HALO^®^ AI, to identify myxoid stroma areas. This network was developed based on the DenseNet-121 [29] deep learning model. Other types of tissue classifications, such as the muscle layers, fat and tumour cells, were also annotated and were used to train the classifier to detect non-myxoid stroma areas. The algorithm performance was evaluated by visual inspection iteratively throughout the algorithm training process. Where misclassification of tissue occurred, additional annotations in these areas were made and added as new training regions for the classifier to learn from. In total, the DenseNet algorithm was trained with 1235 training annotations representing the two classes: myxoid stroma (357 annotations, total area: 17.98 mm^2^) and non-myxoid stroma (878 annotations, total area: 683.80 mm^2^). Training and classification were performed at a 1 μm/px resolution and 215,631 training iterations. The minimum detected object size by the classifier was set to 0.1 mm^2^. The probability threshold was set to 80%.

The performance of the classifier to segment myxoid stroma was assessed using the Dice Coefficient which was defined as Dice score = (2 * true positive area)/((2 * true positive area) + false positive area + false negative area). The true positive, false positive and false negative areas were calculated by applying the trained classifier on 40 unseen H&E images. Twenty images were randomly selected within each DR category (immature DR type: *n* = 20, other DR type: *n* = 20). The myxoid stroma areas present within the unseen immature DR type cases were manually annotated prior to comparing to the automatically identified DR regions of interest and were used as the ground truth (Figure 1).

### 4.4. Automated DR Classification

The invasive tumour front, defined as the tumour periphery close to the non-cancerous surrounding tissue [30], was manually annotated within the HALO^®^ software for all cases (*n* = 528; Figure 2a). The deep learning classifier was then run within an automatically created region with either a 500 or 1000 μm border outward from the invasive tumour front, forming two comparable DR regions of interest, namely Margin 1 and Margin 2, respectively (Figure 2b,c). The areas of myxoid stroma which were automatically identified by the classifier resulted in the creation of three histopathological parameters, namely, total myxoid stroma area, the average myxoid stroma area, and the largest single myxoid stroma area, detected within Margin 1 and Margin 2 of each digitised patient sample. These parameters were exported for statistical analysis.

### 4.5. Survival Analysis

The exported data from the classifier run within the HALO^®^ software were in continuous form. In order to categorise patients into immature or other DR type, the continuous data were binarised according to the optimal cut-off points derived using the maximally selected rank statistic from the R survminer package [31]. These cut-off points were acquired using survival data from the training set (*n* = 396) and were directly applied to the testing set of patients (*n* = 132). The largest single myxoid stroma area was also binarised according to the size of the area as dictated by the manual methodology (area = 0.196 mm^2^), as previously described [6]. The 81 cases which were used in the training and accuracy assessment of the image analysis algorithm were included as part of the training set for the survival analysis.

Univariate Cox regression and Kaplan–Meier survival analysis were performed to assess the prognostic significance of DR both classed manually and by the deep learning classifier on both training and test cohorts using Rstudio [32]. *p* values less than 0.05 were considered statistically significant. Using the Benjamini–Hochberg procedure [33], *p* values from the KM analyses were corrected for false discover rate. Multivariate Cox regression with a forward stepwise method was also performed using SPSS v24 [34]. Disease-specific survival was used for all our survival analysis. This was defined as the length of time (in months) from the date of surgical resection to the date of death from CRC or the last censoring date for surviving patients.

## 5. Conclusions

In conclusion, we present the first study to develop a deep learning algorithm for the standardised and reproducible classification of DR in CRC whole-slide H&E images. Furthermore, we demonstrated that the automatically classed DR is of high prognostic significance. Specifically, the automatically classed DR was superior to the manual DR classification in patient stratification both on the training and a test cohort. The present study therefore suggests that the application of machine learning approaches can help by not only identifying complex patterns present within histopathological images in a standardised and reproducible manner, but also report a more accurate patient stratification. This could, therefore, aid clinical decision making on adjuvant therapy and follow-up, and hence improve the current patient prognosis.

## Figures and Tables

**Table 1:** Patient clinicopathological characteristics for the training and test sets.

Features	Training Set (*n* = 396)	Test Set (*n* = 132)
	Freq. (%)	Freq. (%)
Age		
≤70	247 (62.4)	84 (63.6)
71–79	108 (27.3)	40 (30.3)
≥80	41 (10.4)	8 (6.1)
Gender		
Male	237 (59.8)	78 (59.1)
Female	159 (40.2)	54 (40.9)
pT Stage		
pT3	303 (76.5)	99 (75.0)
pT4	93 (23.5)	33 (25.0)
pN Stage		
pN0	206 (52.0)	74 (56.1)
pN1	132 (33.3)	34 (25.8)
pN2	58 (14.6)	24 (18.2)
Tumour Site		
Left	121 (30.6)	34 (25.8)
Right	114 (18.8)	39 (29.5)
Rectal	161 (40.7)	59 (44.7)
Differentiation		
Moderate	206 (52.0)	59 (44.7)
Poor	25 (6.3)	18 (13.6)
Well	165 (41.7)	55 (41.7)
Tumour Type		
Adenocarcinoma	378 (95.5)	121 (91.7)
Mucinous	18 (4.5)	11 (8.3)
DR type		
Immature	94 (23.7)	31 (23.5)
Other	302 (76.3)	101 (76.5)

Abbreviations: Freq., frequency; DR, desmoplastic reaction.

**Table 2:** Cut-off point values for desmoplastic reaction features derived from image analysis.

Features	Cut-Off Value (mm^2^)
Total myxoid stroma area in Margin 1	0.27392
Average myxoid stroma area in Margin 1	0.00622
Largest single myxoid stroma area in Margin 1 ACP	1.04863
Largest single myxoid stroma area in Margin 1 MCP	0.19600
Total myxoid stroma area in Margin 2	0.31949
Average myxoid stroma area in Margin 2	0.15859
Largest single myxoid stroma area in Margin 2 ACP	0.17410
Largest single myxoid stroma area in Margin 2 MCP	0.19600

Abbreviations: ACP, automatic cut-off point; MCP, manual cut-off point.

**Table 3:** Univariate Cox regression results for the clinicopathological data, and automatic and manual desmoplastic reaction assessment on the training set.

Features	Freq. (%)	Univariate	
		HR (95% CI)	*p*
pT Stage		1.887 (1.091–3.265)	0.023
pT3	303 (76.5)		
pT4	93 (23.5)		
pN Stage		1.795 (1.297–2.484)	<0.001
pN0	206 (52.0)		
pN1	132 (33.3)		
pN2	58 (14.6)		
Differentiation		0.969 (0.744–1.263)	0.816
Moderate	206 (52.0)		
Poor	25 (6.3)		
Well	165 (41.7)		
Tumour Type		2.813 (1.204–6.573)	0.017
Adenocarcinoma	378 (95.5)		
Mucinous	18 (4.5)		
Total myxoid stroma area in Margin 1		2.742 (1.559–4.821)	<0.001
High	197 (49.7)		
Low	199 (50.3)		
Average myxoid stroma area in Margin 1		2.439 (1.293–4.600)	0.006
High	250 (63.1)		
Low	146 (36.9)		
Largest single myxoid stroma area in Margin 1 ACP		2.546 (1.395–4.646)	0.002
Yes	51 (12.9)		
No	345 (87.1)		
Largest single myxoid stroma area in Margin 1 MCP		1.792 (1.071–2.998)	0.026
Yes	169 (42.7)		
No	227 (57.3)		
Total myxoid stroma area in Margin 2		3.527 (1.784–6.973)	<0.001
High	239 (60.4)		
Low	157 (39.6)		
Average myxoid stroma area in Margin 2		2.356 (1.293–4.295)	0.005
High	50 (12.6)		
Low	346 (87.4)		
Largest single myxoid stroma area in Margin 2 ACP		2.941 (1.612–5.367)	<0.001
Yes	216 (54.5)		
No	180 (45.5)		
Largest single myxoid stroma area in Margin 2 MCP		2.671 (1.501–4.752)	<0.001
Yes	207 (52.3)		
No	189 (47.7)		
Manually Assessed DR		2.588 (1.546–4.331)	<0.001
Immature	94 (23.7)		
Other	302 (76.3)		

Abbreviations: Freq., frequency; HR, hazard ratio; CI, confidence interval; ACP, automatic cut-off point; MCP, manual cut-off point; DR, desmoplastic reaction.

**Table 4:** Features entered into the multivariate forward stepwise Cox regression.

Variables in the Equation	Multivariate Cox Regression Model			
	HR	95% CI		*p*
		Lower	Upper	
Total myxoid stroma area in Margin 2	3.527	1.784	6.973	<0.001
pN Stage	1.490	1.062	2.091	0.021
Variables not in equation				
pT Stage				NS
Differentiation				NS
Tumour Type				NS
Manually Assessed DR				NS
Total myxoid stroma area in Margin 1				NS
Average myxoid stroma area in Margin 1				NS
Largest single myxoid stroma area in Margin 1 ACP				NS
Largest single myxoid stroma area in Margin 1 MCP				NS
Average myxoid stroma area in Margin 2				NS
Largest single myxoid stroma area in Margin 2 ACP				NS
Largest single myxoid stroma area in Margin 2 MCP				NS

Significant features are shown on the top half of the table and features which are not significant are shown on the bottom half of the table. Abbreviations: HR, hazard ratio; CI, confidence interval; DR, desmoplastic reaction; ACP, automatic cut-off point; MCP, manual cut-off point.

**Table 5:** Univariate Cox regression results for the total myxoid stroma area in Margin 2, manually assessed DR and the largest single myxoid stroma area in Margin 1 ACP of the test set.

Features	Freq. (%)	Univariate	
		HR (95% CI)	*p*
Total myxoid stroma area in Margin 2		3.743 (1.091–12.830)	0.036
High	82 (62.1)		
Low	50 (37.9)		
Manually Assessed DR		2.635 (1.083–6.408)	0.033
Immature	31 (23.5)		
Other	101 (76.5)		
Largest single myxoid stroma area in Margin 1 ACP		4.654 (1.912–11.330)	<0.001
Yes	21 (15.9)		
No	111 (84.1)		

Abbreviations: Freq., frequency; HR, hazard ratio; CI, confidence interval; ACP, automatic cut-off point; DR, desmoplastic reaction.

## Data Availability

The data and the code used in this study are available from the corresponding author upon request.
